# Hepatitis C Treatment: Trial by Design

**DOI:** 10.4103/1319-3767.39617

**Published:** 2008-04

**Authors:** Khalid I. Bzeizi

**Affiliations:** Division of Gastroenterology and Hepatology, Department of Medicine, Riyadh Military Hospital, Riyadh, Saudi Arabia. E-mail: kbzeizi@rmh.med.sa

Hepatitis C is a major health problem in Saudi Arabia and, as is the case, in most parts of the world. The standard treatment of all genotypes of hepatitis C consists of pegylated interferon (IFN) alfa and ribavirin combination therapy. Majority of hepatitis C viruses (HCV) patients in the Gulf region and the Middle East in general are infected with Genotype 4. Genotype 4 can be singled out as the one least studied among all the other genotypes. This is not entirely surprising considering that research is not a priority, to put it mildly, in the Middle-East. In this issue of the Saudi Journal of Gastroenterology, Al Ashgar and colleagues report the treatment outcome of 335 HCV patients treated with peginterferon α-2a and ribavirin combination therapy.[1] The study is retrospective; however, it was well conducted and covered the essentials expected in a well-designed study. The protocol of management was well defined and the patients were managed and monitored closely according to the standardized protocol.

The study is designed to assess the efficacy and safety of 48 weeks peginterferon α-2a and ribavirin combination therapy. The authors also looked at the predictors of sustained virologic response (SVR) in their cohort of 335 consecutive Saudi patients with HCV infection treated in their center. Eighty seven percent of patients completed treatment and about 55% achieved SVR. On intention-to-treat analysis, the overall number of patients with SVR was about 48%. The divergence of the study's findings from the prospective studies published earlier for genotype 4 lies mainly in the overall SVR.[2,3] The authors of the study have indicated that the reason for lower SVR than other gentotype 4 studies is the fact that they have included patients with co-morbidities, non-responders to previous IFN treatment organ transplant patients, and those with co-infection with HBV or HIV infections. It is difficult to ascertain how much the design of the study could have impacted on the relatively low SVR. As with any retrospective and post-marketing study, the level of motivation and compliance of patients is known to be relatively lower than in patients recruited for prospective studies. A more plausible explanation for the low SVR could be the difference of genotype 4 subtypes. The largest number of studies in genotype 4 infected patients came from Egypt where the SVR was generally higher than the findings of this study. Genotype 4a is the most common in Egypt unlike Saudi Arabia where genotypes 4c/4d are the most prevalent as found by an epidemiological study by Shobokshi *et al*.[4] Roulot *et al.*[5] analyzed epidemiological features and SVR rates in a retrospective study of 1532 HCV-4-infected patients, including 1056 patients infected in France, 227 immigrants infected in Egypt, and 249 in sub-Saharan Africa. The 4a subtype was largely predominant (93%) among patients infected in Egypt. SVR rates were higher in patients infected in Egypt, compared with those infected in France or Africa (54.9%, 40.3%, and 32.4%, respectively; *P* < 0.05). An overall better response was observed in patients infected with the 4a subtype compared with 4d subtype-infected individuals. Patients in the French group infected with the 4a subtype showed SVR rates similar to those of patients in the Egyptian group.

Another interesting finding of the study worth mentioning here is the SVR (34.6%) of patients who previously received conventional IFN therapy. SVR was better than the results of many studies in both genotype 1 and genotype 4 and the authors attributed this to a potential immunological mechanisms.[6,7] The degree of fibrosis as well as viral load did not appear to be predictive of response to treatment, findings that again differ from what has been established from other studies particularly the prospective ones. The time lag from taking the biopsies and the initiation of treatments was listed as one of the explanation for lack of difference in SVR according to the degree of fibrosis. It remains, however, difficult to come with plausible explanations for such divergence from the findings of other studies except perhaps for the fact that this study is a retrospective one. Nevertheless, the study is an important addition in this region towards the management of HCV patients.

The fact remains that the overall SVR particularly for genotypes 1 and 4 is sub-optimal as this study and similar ones have shown. Taking a lead from this study, there is a need for optimizing the use of existing agents and tailoring the treatment to the individual patients in order to maximize the SVR. The peginterferon-based treatment for HCVwill be with us for the foreseeable future and therefore, randomized multicenter trials, particularly for HCV genotype 4, are still needed utilizing this treatment modality. Clinical trials should aim to study aspects such as treatment duration, optimum ribavirin dosing, and utility of RVR. Likewise, every effort should be made every effort should be made to explore the place of the newer class of agents, the small molecules that are viral enzyme inhibitors in treatment of genotype 4 patients. The protease enzyme inhibitors appeared to be the most promising in recent phase II/III trials for genotype 1. Such studies should be extended to include genotype 4 patients who are equally difficult to treat. The resources available in this region certainly match the huge scale of disease burden and those of us ideally positioned should take the lead in setting up relevant clinical trials as early as possible.
